# Myiasis

**DOI:** 10.1002/ccr3.2711

**Published:** 2020-02-05

**Authors:** Seerwan Qaradaghy, Sirwan Hadad

**Affiliations:** ^1^ Department of Breast and Plastics Sheffield Teaching Hospitals NHS Foundation Trust Sheffield UK

**Keywords:** breast cancer, diabetes, maggot, Myiasis, poor hygiene

## Abstract

Late presentation of breast cancer is more likely to be complicated and fatal. Local invasion, tissue destruction, skin lose, and superadded infection/infestation make surgical intervention very challenging.

## Graphical abstract

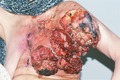

Q: What is this condition?

A: Maggot infestation of a fungating left breast carcinoma, or what is called Myiasis. This self‐neglected middle‐aged woman presented with this rare condition and died few weeks later Figure 1.^1^ Wound debridement and symptom control were the mainstay of treatment. Approximately 50 maggots of the Wohlfahrtia Magnifica type were removed.

**Figure 1:**
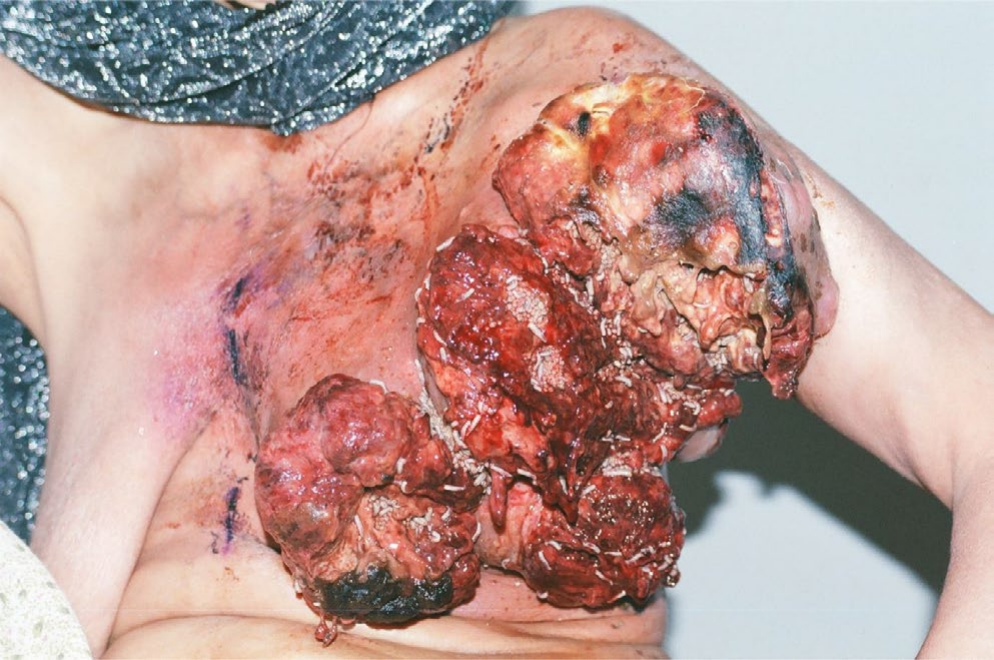
Fungating left breast carcinoma with skin necrosis, ulceration, and infestation by maggots

This parasitic larva of fly lives on the debris and necrotic tissue. Diabetes, immunosuppression, and poor hygiene,^2^ in a hot climate and tropical countries are the main risk factors. Management is wound debridement and disinfection along with controlling the risk factors and treating the underlying cause.

## AUTHORS' CONTRIBUTIONS

Dr SQ: contributed to this work by obtaining consent, organizing clinical photography, writing, and reviewing the manuscript. Mr SH: contributed to this work by writing and reviewing the manuscript.
